# Primary Pituitary Lymphoma in Immunocompetent Patients: A Report on Two Case Studies and the Review of Literature

**DOI:** 10.3389/fendo.2020.562850

**Published:** 2021-02-04

**Authors:** Lian Duan, Jie Liu, Yan Zhang, Lijia Cui, Xiao Zhai, Boju Pan, Lin Lu, Hui Pan, Yong Yao, Huijuan Zhu

**Affiliations:** ^1^ Key Laboratory of Endocrinology of National Health Commission, Department of Endocrinology, Peking Union Medical College Hospital, Chinese Academy of Medical Science and Peking Union Medical College, Beijing, China; ^2^ Department of Neurosurgery, Peking Union Medical College Hospital, Chinese Academy of Medical Science and Peking Union Medical College, Beijing, China; ^3^ Department of Hematology, Peking Union Medical College Hospital, Chinese Academy of Medical Science and Peking Union Medical College, Beijing, China; ^4^ Department of Pathology, Peking Union Medical College Hospital, Chinese Academy of Medical Science and Peking Union Medical College, Beijing, China

**Keywords:** primary pituitary lymphoma, large B-cell lymphoma, sellar mass, primary CNS lymphoma, radiotherapy, chemotherapy

## Abstract

Primary pituitary lymphoma (PPL) represents an extremely rare entity. Here, we have reported two recently identified cases of immunocompetent PPL having diffuse large B-cell lymphoma by surgical biopsy. Both patients had hypopituitarism, with one patient developing right ptosis. In both patients, MRI and FDG-PET/CT depicted sellar mass that extended into the cavernous sinus with the right sphenoid also present in one of the patients. No systemic disease was found in these two patients. Surprisingly, we found that both patients had infiltrative lesions in sphenoid sinus mucosa pathologically, but the sphenoid bones that composed the sellar base were visually intact during the biopsy procedure. Chemotherapy was administered to both patients, where one patient achieved remission at the recent follow-up, whereas the other one did not respond to the treatment. The diagnosis of PPL is usually difficult if solely dependent on history, clinical presentation, biochemical indexes, and radiographic findings. We have also updated and reviewed the epidemiologic features, clinical presentations, pathological characteristics, potential mechanisms, therapeutic orientation, and prognostic advances of PPL. A total of 40 cases (including ours and four pediatric patients), histologically diagnosed, were analyzed in terms of clinical presentation, endocrine abnormality, radiological features, pathology, treatment, and follow-up. Hypopituitarism and headache were the most common presentation of PPL, while diabetes insipidus was reported in 13 patients (43.3%). B cell lymphoma was the most common type of pathology, followed by T-cell and NK/T cell. PPL was more invasive in nature at the suprasellar region (72.5%), cavernous sinus (52.5%), and sphenoidal sinus (27.5%) in 29, 21, and 11 patients, respectively. Pediatric patients with PPL seem to be different compared to their adult counterparts in terms of pathogenesis, clinical presentation, and radiological features. The management of PPL usually follows the treatment protocols for PCNSL but has a poor prognosis compared to the pituitary involvement of systemic lymphoma.

## Introduction

Primary pituitary lymphoma (PPL) is an extremely rare clinical entity with only 40 immunocompetent cases identified and reported to date. It is commonly considered to be a diffuse lymphoma that is limited to the sellar or parasellar regions without any evidence of systemic involvement. The disease shows a slight but not significant female prevalence with an onset at around the age of 60 years (1–3). Regarding the histology, a large majority are of B cell origin compared to T cell or NK/T cell origin, as in the case for primary CNS lymphoma (3, 4). The clinical presentations are always atypical, including headache, hypopituitarism, diplopia, hemianopia, and fever (5). The PPL prognosis is usually poor, which emphasizes the importance of earlier detection and management to improve the outcomes (6). Here, we have reported two recently diagnosed cases of PPL with a comprehensive review of our current understanding of PPL. We also concluded that PPL tends to spread to the sphenoidal area through vascular and bony approaches, which might be a distinct entity from primary CNS lymphoma.

## Methods

**Figure 1:**
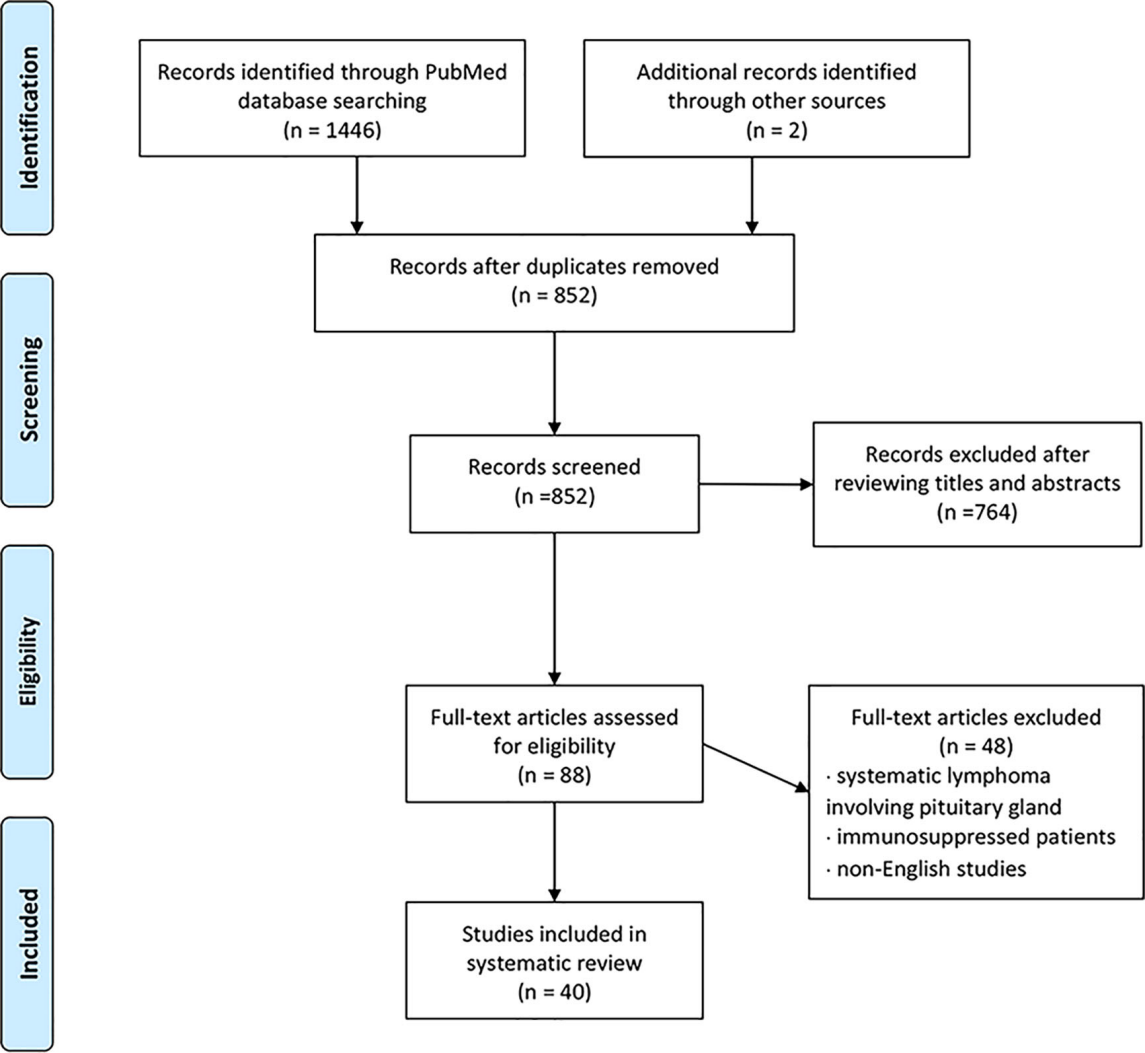
Flowchart of study selection using the PRISMA guidelines.

We conducted a literature review using the PubMed database. Keywords of “pituitary lymphoma”, “pituitary tumor”, and “lymphoma” were used to select adequate papers till May 2020 where the diagnosis of PPL was confirmed histologically. PPL caused by systemic lymphoma or the ones that occurred in immunosuppressed patients were excluded. Only studies in English were considered. Also, we have reviewed references of relevant studies. Some detailed information that was not provided by the original papers was again referred through the latest update (2), acquired through the e-mail contact with the corresponding authors of each article. A total of 36 adult cases (**Table 1**) with four pediatric cases (**Table 2**) were reviewed in terms of clinical presentation, endocrine abnormality, radiological features, pathology, treatment, and follow-up (**Figure 1**). Firstly, we present our two cases.

**Table 1:** The clinical summary of 36 immunocompetent adult PPL patients.

Authors and year	Ref. no	Gender/age	Clinical presentation	Endocrine abnormality	Radiological features	Pathology	Treatment	Follow up
Singh et al., 1993	(7)	M 28	Headache, blurred vision, bitemporal hemianopia	NM	Intrasellar and suprasellar mass extending to the cavernous sinus and sphenoidal sinus with bony destruction	Primary non-Hodgkin lymphoma	Surgery, CHT(CHOP), RT (5000 rads)	Remission at six months
Samaratunga et al., 1997	(8)	M 66	Presyncopal episodes, dizziness, anorexia, nausea, and weight loss	Anterior hypopituitarism	Intrasellar non-enhancing mass	Diffuse large B-cell lymphoma	Surgery, RT (4800 rads)	Clinically stable and radiological regression at eight months, died of myocardial infarction
Shaw et al., 1997	(9)	F 73	Diplopia, fatigue, headache, thirst, and polyuria.	Panhypopituitarism, hyperprolactinemia	Intrasellar and sphenoidal mass, thickening of the pituitary stalk, enhancing mass in the floor of the third ventricle	Non-Hodgkin lymphoma.	Radical RT (4000 cGy in 20 fractions)	Regression at 21 months after RT
Kuhn et al., 1999	(10)	F 67	Bitemporal hemianopsia, visual acuity deterioration	Panhypopituitarism	Intrasellar, suprasellar, and right parasellar lesion that had invaded the right cavernous sinus	Mixed lymphoma (lymphoblastic T cell) and adenoma (approximately 50% of cells were positive for FSH)	Surgery, fractionated RT	Some residual tumor within the right cavernous sinus at the end of RT (5 months after surgery)
Freda et al., 1999	(11)	M 50	Retro-orbital pain (2), cranial neuropathy	NM	Parasellar cavernous sinus lesion extends to sellar	B cell lymphoma (2)	NM	NM
Mathiasen et al., 2000	(12)	M 65	Decreased libido, fatigue, muscle weakness, mild dyspnea on exertion	Anterior hypopituitarism, hyperprolactinemia	Diffusely enlarged homogeneous enhancing sellar lesion	Diffuse large-cell malignant lymphoma, B-cell type	Surgery, CHT (Cytoxan, vincristine, bleomycin, and adriamycin)	Favorable outcome at six months (2)
Au et al., 2000	(13)	M 82	Headache, blindness, hemianopia, and papilledema	Panhypopituitarism	heterogeneously isointense bilobed tumor enlarging the pituitary fossa, hypothalamus, and optic chiasm were severely compressed	Diffuse large B cell lymphoma (CD20 positive) mixed with adenoma (weak FSH positive)	Field RT	NM
Landman et al., 2001	(14)	F 86	Fever, weight loss	Partial anterior hypopituitarism	Sellar mass within a minimally enlarged pituitary fossa	B-cell lymphoma	Surgery, CHT	Died three months after diagnosis
Lee et al., 2002	(15)	M 42	Blurred vision, loss of libido, temporal visual-field defect	Panhypopituitarism, slight hyperprolactinemia	Sellar mass with supra- and parasellar extension, focal bony erosion due to the mass on the left side of the sphenoid body	Low-grade MALT lymphoma, B cell type	Surgery, systematic CHT	Grossly free of symptoms for the six months of clinical follow-up
Kaufmann et al., 2002	(16)	M 74	Seizure, weakness, dizziness and falling, weight loss, thirst, cold intolerance, worsening constipation, impotence, possible mild bitemporal visual field deficit.	Anterior hypopituitarism	Sellar and suprasellar mass, erosion of the left sellar floor	Large B-cell lymphoma	Surgery, 6-wk 4600-cGy course of conventional RT to the pituitary	Died soon after RT
Kaufmann et al., 2002	(16)	M 65	Diplopia, right retrobulbar pain, decreased energy and libido	Anterior hypopituitarism	Sellar and suprasellar mass extending into the right cavernous sinus, sphenoid sinus erosion	High-grade, large B cell lymphoma	Surgery, stereotactic radiosurgery, 4 cycles of Codox-M CHT	Died of pulmonary failure 2 y after surgery
Katz et al., 2003	(17)	F 64	Headache, nausea, vomiting, polyuria, binocular diplopia, ptosis of the eyelid	Panhypopituitarism, hyperprolactinemia	Enlargement of the pituitary gland, cavernous sinus thrombosis, invasive process involving the cavernous sinus, skull base, and clivus with extradural extension	Diffuse, large B-cell lymphoma	CHT (6 cycles of CHOP regimen complemented by intrathecally administered MTX), 2,000 cGy of external beam RT to the clivus and cavernous sinus in fractionated doses	Remission for a while but died of pneumonia three years later
Huang et al., 2005	(18)	M 47	Fever, decreased libido, ptosis	Partial anterior hypopituitarism	Homogenous enhanced pituitary mass	T-lymphoid cells concomitant presence of some B-lymphoid cells	Systematic CHT with CHOP regimen, intrathecal MTX injection, RT	Significant tumor reduction for five months
Liu et al., 2007	(3)	M 26	Headache, nausea, vomiting, diplopia, fever	Partial anterior hypopituitarism, hyperprolactinemia	Enhancing sellar mass with suprasellar extension compressing the optic chiasm and involvement of planum sphenoidal, anterior clinoid process, infundibulum, clivus, and cavernous sinus	Extranodal NK/T cell lymphoma	Fractionated intensity-modulated RT (25 fractions of 200 cGy for a total dose of 5000 cGy), 6 cycles of hyper-CVAD CHT (cyclophosphamide, vincristine, doxorubicin, and dexamethasone) with intrathecal MTX and cytarabine	Died of systemic progression six months later
Rudnik et al., 2007	(19)	M 37	Headache, worsening of visual acuity, bilateral blurred vision, bitemporal hemianopia	None	A large intrasellar mass with a widening of the sella turcica, penetrating backward into the anteropontal cisterns, which were narrowed, upward enclosing the hypothalamic infundibulum and optic chiasm, cavernous sinus and sphenoidal involvement	Large B-cell lymphoma	Surgery, 6 cycles of CHT with the CHOP protocol, RT (whole brain 40 Gy and a boost to the tumor 50 Gy)	A stable remnant of the tumor and only a stable, slight visual-field defect at 52 months’ follow up
Chen et al., 2008	(20)	F 63	left retro-ocular pain, double vision, fever	NM	An irregular enhanced lesion in the sellar region involving the sphenoid sinus, cavernous sinus, and clivus, with sellar floor destruction	Diffuse large B-cell lymphoma	Surgery, focal RT, and systemic CHT	liver and lung metastasis and died seven months after the initial diagnosis
Romeike et al., 2008	(21)	F 64	Headache, double vision. ptosis, bitemporal hemianopia	Panhypopituitarism (previously identified)	A contrast-enhancing intra- and suprasellar mass	Recurrent pituitary adenoma (5% positive for FSH) tightly admixed with lymphoma (T cell)	Surgery, 6 cycles of MTX and external beam radiation of the head with 45 Gy	Stable residual tumor at 19 months
Kozakova et al., 2008	(22)	F 60	bitemporal visual field deficit, headache, polyuria, polydipsia, fever	Panhypopituitarism, hyperprolactinemia	Sellar mass with suprasellar extension	High-grade B-cell non-Hodgkin lymphoma of the Burkitt type	NM	NM
Wolfe et al., 2009	(23)	F 45	Headache, dizziness, blurred vision, bitemporal hemianopsia, ptosis, face numbness	Partial anterior hypopituitarism, hyperprolactinemia	Heterogeneously enhancing, sellar–suprasellar mass with impingement on the optic nerves and chiasm, and involvement of the cavernous sinuses as well as thickening of the pituitary stalk	B cell lymphoma	Surgery, CHT (MTX, vincristine, procarbazine, and dexamethasone), RICE, autologous transplant (2)	Multiple relapses at one year and two years Autologous stem cell transplant at two years with good evolution (2)
Moshkin et al., 2009	(24)	M 62	Headache, left sixth cranial nerve palsy, diplopia	None	Aggressive osteolytic lesion of the sellar and clivus, with the involvement of the basiocciput, right carotid artery, and the right and left pterygopalatine fossae	Diffuse large B cell lymphoma	NM	NM
Carrasco et al., 2010	(25)	F 49	Weakness, headache, asthenia, weight loss, increase in thirst, polyuria, cold intolerance, abdominal pain, nauseous, and dizziness, later diplopia	Panhypopituitarism, hyperprolactinemia	Sellar and suprasellar enhancing mass with thickening of the pituitary stalk and chiasmal compression, later infiltration of the left cavernous sinus	lymphoplasmacytic B cell lymphoma (low grade)	Tumoral resection but only a biopsy of the pituitary mass, CHT (iv and intrathecal MTX, dexamethasone, cytarabine), fractionated conformational RT of 30 Gy	Remission at four years after surgery
Li et al., 2010	(5)	F 41	Polydipsia and polyuria, galactorrhea amenorrhea syndrome	Partial anterior hypopituitarism, hyperprolactinemia	Intrasellar and suprasellar mass with thickening of the pituitary stalk, obstructive hydrocephalus	High-grade, large B cell lymphoma	Surgery, RT (25 fractions of 200 cGy for a total dose of 5000 cGy), and CHT with intrathecal MTX	No recurrence for seven months after surgery
Bayraktar et al., 2010	(26)	F 47	Headache, blurry vision, bitemporal hemianopsia	Hyperprolactinemia	Sellar mass with partial encasement of the right internal carotid artery within the cavernous sinus	Diffuse large B cell lymphoma	Surgery, DeAngelis CHT protocol (methotrexate, vincristine Procarbazine, cytarabine), R-CHOP, R-ICE, prophylactic intrathecal MTX, autologous stem cell transplantation	Multiple relapses during CHT, clinical remission eight months after stem cell transplantation.
Hayasaka et al., 2010	(27)	M 71	Appetite loss and sudden visual loss	Hyperprolactinemia	Intrasellar and suprasellar mass that was moderately and homogeneously enhanced, focal moderate FDG uptake only in the pituitary gland	Non-Hodgkin lymphoma (CD20+, CD5-, CD10-, BclII+, Bcl6-)	NM	NM
Martinez et al., 2011	(28)	F 71	Facial palsy, bilateral temporal hemianopsia, syncopal spells, acromegaly	Increased level of IGF-1 and GH	well-circumscribed mass extending into the suprasellar cistern and compressing the optic chiasm with penetration into the right cavernous sinus	Large B-cell lymphoma, adenoma, and lymphocytic hypophysitis	Surgery, a 5040 cGy course of intensity-modulated RT	Remission at one year
Papanastasiou et al., 2012	(29)	F 60	Muscle weakness, headache, and eyelid ptosis	Partial anterior hypopituitarism	Pituitary sellar mass extending into the suprasellar region, compressing the optic chiasm and invading the left cavernous sinus	Diffuse large B-cell non-Hodgkin’s lymphoma	Surgery, systemic CHT with intraspinal MTX infusion, and the R-MPV regimen	Died of a hospital-acquired infection at two months
Rainsbury et al., 2012	(30)	F 67	Headache, left visual field defect	None	Pituitary tumor extending inferiorly to occupy the sphenoid sinus	Diffuse, large, high-grade B-cell pituitary lymphoma	Surgery, 4 cycles of CHT and stereotactic RT	No recurrence at 15-month follow up
Wiens et al., 2012	(31)	F 58	Headache, right-sided ptosis, fatigue, vision loss, increased thirst and urine output, altered mental status, weight loss, slurred speech	Partial anterior hypopituitarism	Large, heterogeneously enhancing intrasellar lesion and a much larger suprasellar component extending to the right cavernous sinus	T-cell lymphoblastic lymphoma with corticotrophic cell hyperplasia	Surgery, intrathecal CHT, RT (craniospinal radiation was administered (18 fractions of 200 cGy for a total dose of 3600 cGy), boosted with an additional 1080 cGy to the pituitary fossa and 5040 cGy to the lower spine)	leptomeningeal carcinomatosis one week after surgery
Wilkle et al., 2012	(32)	F 59	Headache, diplopia	Partial anterior hypopituitarism, hyperprolactinemia	Enhancing sellar mass extending to the suprasellar cistern and along the pituitary stalk and cavernous sinuses	High-grade B-cell non-Hodgkin’s lymphoma	None	Died before the therapy initiated
Tarabay et al., 2016	(2)	M 73	Headache, diplopia, retro-orbital pain	DI	Sellar mass extending to the suprasellar cistern, cavernous sinus, and orbital apex in contact with optic chiasm and optic nerve	Diffuse large B-cell lymphoma	Surgery, CHT (R -CHOP, high dose MTX)	Clinical remission and radiological improvement at 15 months
Ravindra et al., 2017	(33)	F 61	Headache, acromegaly	Partial anterior hypopituitarism, hyperprolactinemia, elevated GHRH and IGF-1	Isointense, homogenously enhancing sellar mass with no evidence of pituitary stalk displacement	Diffuse large B-cell lymphoma, somatotroph hyperplasia	3 cycles of R-CHOP followed by 3600 cGy of stereotactic RT with 180 cGy for 20 fractions over 27 days	Radiological remission at eight months after initial pathological diagnosis (2 months after completion of RT), normalized IGF-1 within five months of CHT and RT
Ban et al., 2017	(34)	M 74	Bilateral retro-orbital pain, headaches, nausea, left-sided ptosis, diplopia	Partial anterior hypopituitarism, hyperprolactinemia, elevated IGF-1	Soft tissue mass in the pituitary fossa with suprasellar extension and extension to sphenoidal sinus and cavernous sinus	Concomitant primary pituitary lymphoma (diffuse large B-cell non-Hodgkin’s lymphoma) and FSH adenoma	Surgery, 4 cycles of CHT (R-CHOP with intrathecal methotrexate over about eight weeks and G-CSF support for six days)	Remission for over 32 months
Gupta et al., 2017	(35)	F 55	Headache, diminution of vision, and recent-onset altered sensorium	Partial anterior hypopituitarism, hyperprolactinemia	A mass lesion in the sellar-suprasellar region with non-visualization of pituitary gland separately, extending to involve suprasellar cistern, lateral ventricle, cavernous sinus, optic chiasm, and infundibulum	Pituitary T cell lymphoma concurrent with ACTH and TSH positive pituitary adenoma	Surgery	Died two months after the surgery
Velho et al., 2018	(36)	M 23	Headache, diminution of vision, weakness, easy fatiguability, vomiting, and episodes of hypoglycemia	Anterior pituitary hypofunction	A large, ill−defined irregular heterogeneously enhancing mass lesion in the sellar-suprasellar region displacing the optic chasm upward along with the involvement of the clivus	NK/T cell lymphoma	Surgery, CHT	Doing well at 1-year follow-up
Our case 1		M 61	Headache, right ptosis, blurred vision, weakness, and anorexia	Partial anterior hypopituitarism, hyperprolactinemia	A sellar mass extending to the cavernous sinus and right sphenoid	Diffuse large B-cell lymphoma	CHT (rituximab, MTX, lenalidomide) and intrathecal dexamethasone and cytarabine	Alleviation of headache, improvement of ptosis, and reduction of mass in size after 2 courses of CHT
Our case 2		F 65	Headache, nausea, vomiting, thirst, polydipsia, polyuria, anorexia, weakness	Panhypopituitarism	A soft tissue mass in the sellar region which was not distinguished from the optic chiasm	Diffuse large B-cell lymphoma	CHT	Obvious enlargement of the sellar mass in three months after diagnosis and died eight months after diagnosis.

CHOP, cyclophosphamide–doxorubicin–vincristine–prednisolone; CHT, chemotherapy; DI, diabetes insipidus; F, female; G-CSF, granulocyte-colony stimulating factor; M, male; MTX, methotrexate; NM, not mentioned; R-CHOP, rituximab–cyclophosphamide–vincristine–adriamycin–prednisone; R-MPV, rituximab–methotrexate–vincristine–procarvazine; RICE, rituximab–ifosfamide–carboplatin–etoposide; RT, radiotherapy.

**Table 2:** The clinical summary of 4 pediatric immunocompetent PPL patients.

Authors and year	Ref. no	Gender/age	Clinical presentation	Endocrine abnormality	Radiological features	Pathology	Treatment	Follow up
Silfen et al., 2001	(37)	M 15	Polydipsia, polyuria, and weight loss	Panhypopituitarism	Enhancing lesion in the region of the pituitary stalk	High-grade malignant B-cell lymphoma	CHT (high-dose MTX and cytarabine, prednisone, vincristine, cyclophosphamide, doxorubicin, and hydrocortisone)	Remission for 17 months
Baleydier et al., 2001	(38)	M 9	Diplopia, signs of meningeal irritation, weight loss, limb muscle weakness	DI	Enhancing infundibular stalk thickening and a nodular hypothalamic mass	Large B-cell lymphoma (identified by CSF cells)	CHT (protocol LMB 96 of the SFOP)	Complete remission for a year after diagnosis
Capra et al., 2004	(39)	F 14	Short stature	GH deficiency	Enhancing and thickened pituitary stalk with the absence of the normal bright signal in the posterior pituitary	B-cell large cell lymphoma	Intravenous and intrathecal chemotherapy alone as per the FAB-LMB 96 (group C) protocol	Complete clinical remission ten months from diagnosis
Huang et al., 2013	(40)	F 7	DI, later panhypopituitarism	Panhypopituitarism	Diffuse thickening of the pituitary stalk with extension to the hypothalamic region, a small, nodular, heterogeneous enhancement of the pineal gland	Diffuse large B-cell lymphoma	CHT	The disappearance of tumor after 16 months of biopsy with persistent panhypopituitarism

CHOP, cyclophosphamide–doxorubicin–vincristine–prednisolone; CHT, chemotherapy; DI, diabetes insipidus; F, female; G-CSF, granulocyte-colony stimulating factor; M, male; MTX, methotrexate; NM, not mentioned; R-CHOP, rituximab–cyclophosphamide–vincristine–adriamycin–prednisone; R-MPV, rituximab–methotrexate–vincristine–procarvazine; RICE, rituximab–ifosfamide–carboplatin–etoposide; RT, radiotherapy.

### Case Presentation

#### Case 1

A 61-year-old male was presented to our clinic reporting episodes of headache and hypophyseal mass for nine months and right eyelid ptosis for four months. In January 2019, he started with an intermittent headache, blurred vision, weakness, and anorexia. He was then admitted to a local hospital for further examination. His serum sodium level was 127 mmol/L with normal blood pressure. The biochemical indexes indicated that he suffered from hypopituitarism. The symptoms of diabetes insipidus were not observed. Pituitary MRI showed a suspicious low signal in the right-wing of the pituitary. Further, he had a history of partial thyroidectomy ten years ago due to a thyroid nodule, with unknown postoperative pathological results. Based on the above evidence, an initial diagnosis of autoimmune hypophysitis was made. The patient then started the replacement therapy using 30 mg hydrocortisone per day and Euthyrox at 50 µg qd, which alleviated headache and improved the right eye acuity.

Five months later, the patient developed sudden right eyelid ptosis. A new MRI disclosed that the pituitary mass had invaded the right cavernous sinus. Glucocorticoid impact therapy with an initial dose of 500 mg qd was initiated, which improved his right eyelid ptosis on the second day of therapy. Nevertheless, a relapse of ptosis occurred three months later and another MRI revealed a 1.3 × 0.9 cm sellar mass with extension to the right cavernous sinus and internal carotid artery. Immunology tests, including ANA, ANCA, serum IgG4, and ACL were reported negative. Lumbar puncture and thoracoabdominal CT scan did not show any remarkable findings. The diagnosis of autoimmune hypophysitis could not be excluded and the cortisol treatment was continued (prednisone 50 mg qd) in addition to 75 mg azathioprine per day.

**Figure 2:**
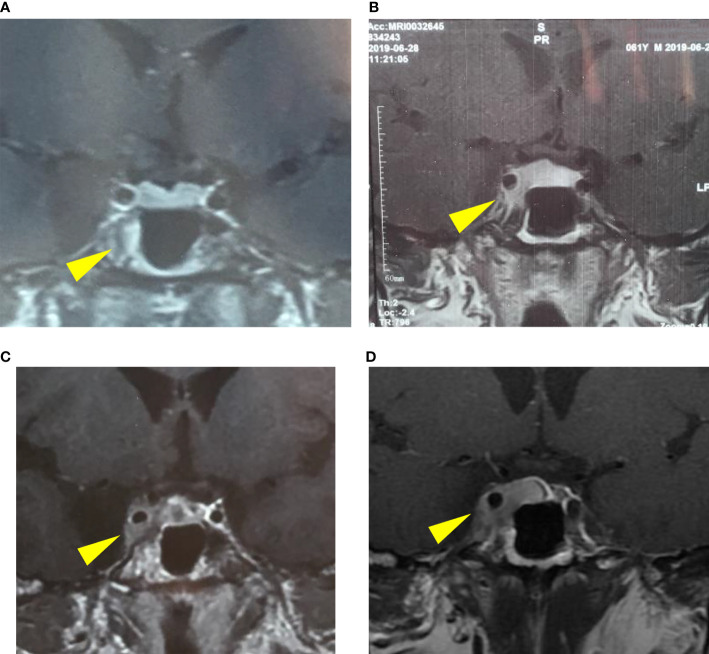
The progression of the pituitary lesion in MRI: **(A)** a suspicious low signal in the right-wing of the pituitary (January 15, 2019), **(B)** enlargement of the pituitary lesion involving right cavernous sinus (June 28, 2019), **(C)** involvement of the right cavernous sinus and internal carotid artery (September 24, 2019), **(D)** a possible macroadenoma, Knosp IV (October 17, 2019).

In October 2019, he was referred to our clinic with several tests, including an electrolyte, serum and urine osmolality, sex hormone (FSH, LH, E, P, T, PRL), IGF-1, thyroid (TSH, FT_3_, FT_4_), immunology (IgG_4_, ANCA, sACE, ESR) and tumor markers tests (CEA, AFP, βhCG), which were all within the normal range. The MRI indicated a possible sellar macroadenoma (12.7 × 6.7 × 11.1 mm) with Knosp IV; the progression of the sellar lesion is shown in **Figure 2**. Physical examination revealed truncal obesity and diabetes mellitus due to glucocorticoid treatment. His right eye showed ptosis with loss of pupil’s direct/indirect light reflex and ocular motility. FDG-PET/CT revealed a hypermetabolic lesion in the right sellar with infiltration to the right sphenoid sinus, suggesting invasive pituitary adenoma. The systemic disease was, therefore, excluded.

**Figure 3:**
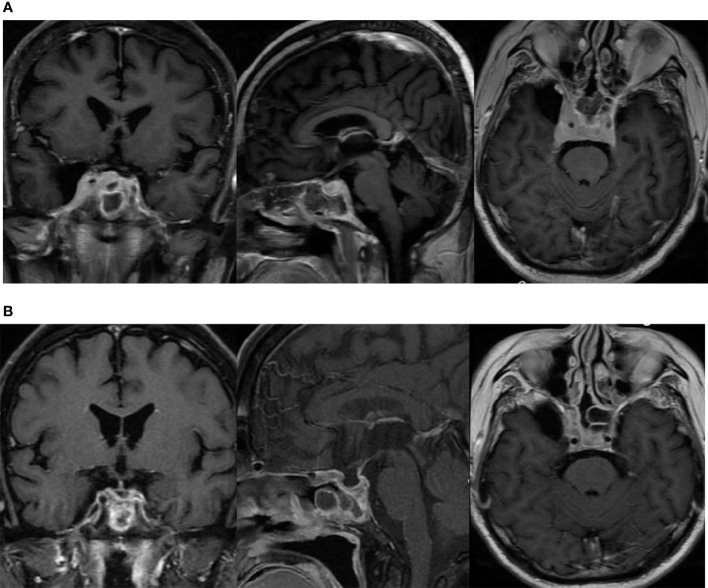
The lesion in the sellar region involving bilateral cavernous sinus as shown in MRI. **(A)** before chemotherapy; **(B)** after two courses of chemotherapy (R^2^-MTX).

The patient underwent a sellar biopsy using an endonasal transsphenoidal approach and the mass on the right side of the sellar displayed a grayish-white hard tissue that looked similar to fish-meat with insufficient blood supply on tissue modality. Pathological analysis established the diagnosis of diffuse large B-cell lymphoma. Cell immunohistochemistry was positive for p53, Bcl-2, Bcl-6, C-MYC, CD20, and CD5, whereas the index for pituitary hormones and epithelial cells tested negative. Cell proliferation index Ki-67 was found to be 70%. Fluorescent *in situ* hybridization (FISH) of EBER ISH was negative, and no abnormality was found in CSF and bone marrow. The diagnosis of primary pituitary lymphoma was eventually established, and the patient received chemotherapy (rituximab 800 mg iv d1, MTX 7 g iv 4 h d2, lenalidomide 25 mg po d1–14) with 5 mg intrathecal dexamethasone and 50 mg cytarabine. After two courses of chemotherapy (R^2^-MTX), the patient achieved remission from prolonged headache and ptosis. MRI confirmed a significant reduction in the size of the sellar mass (**Figure 3**).

#### Case 2

A 65-year-old woman was presented to our clinic with complaints of headache, nausea, and vomiting. Two months earlier, she had developed symptoms of intermittent headache and nausea with no obvious precipitating factors. Then she gradually had thirst, polydipsia, polyuria, anorexia, and weakness. The biochemical indexes measurement showed values indicative for anterior hypopituitarism, with FT_4_ of 8 pmol/L, TSH of 0.11 µIU/ml (normal range is 0.380–4.340 µIU/ml), LH< 0.01 mIU/ml (normal range is 1.24–8.62 mIU/ml), FSH of 1.16 mIU/ml (normal range is 1.27–19.26 mIU/ml), ACTH (8 AM) of 1.91 pmol/L (normal range is 2.2–17.6 pmol/L), serum cortisol (8 AM) of 0.536 µg/dl (normal range is 4.0–22.3 µg/dl), and IGF-1 of 150 ng/ml (normal range is 94–252 ng/ml). She had hypernatremia (serum sodium of 150 mmol/L), increased plasma osmolality (304 Osm/kgH_2_O), and hyposthenuria (98 Osm/kgH_2_O). The examination of the visual field and visual acuity were found normal, although the MRI revealed a 1.5 × 1.3 × 2.2 cm soft tissue mass in the sellar region, which had no defects in the optic chiasm. The PET/CT identified a pituitary mass with a high intake of FDG in the sellar region and had no other remarkable findings. Replacement therapy with desmopressin, prednisone acetate, and Euthyrox was then administered, which provided the remission from thirst, but headache continued with intermittent nausea and vomiting. In December 2019, the patient underwent a sellar biopsy using an endoscopic transsphenoidal approach. Pathological analysis revealed diffuse large B-cell lymphoma. Immunohistology studies showed CD3, CD5, CD20, Bcl-2, Bcl-6, C-MYC, PAX-5, Mum-1, and p53 to be positive. Cell proliferation index Ki-67 was 90%. Fluorescent *in situ* hybridization (FISH) for EBER ISH was negative. Therefore, the patient was diagnosed with primary pituitary diffuse large B-cell lymphoma. The patient then received chemotherapy in her local hospital. However, in February 2020, a recent MRI revealed an obvious enlargement of the sellar mass compared to the former one, which may indicate the patient’s insensitivity to chemotherapy. Our latest follow-up revealed that the patient died in July 2020, which is eight months after the diagnosis.

## Results

### Age and Sex

**Figure 4:**
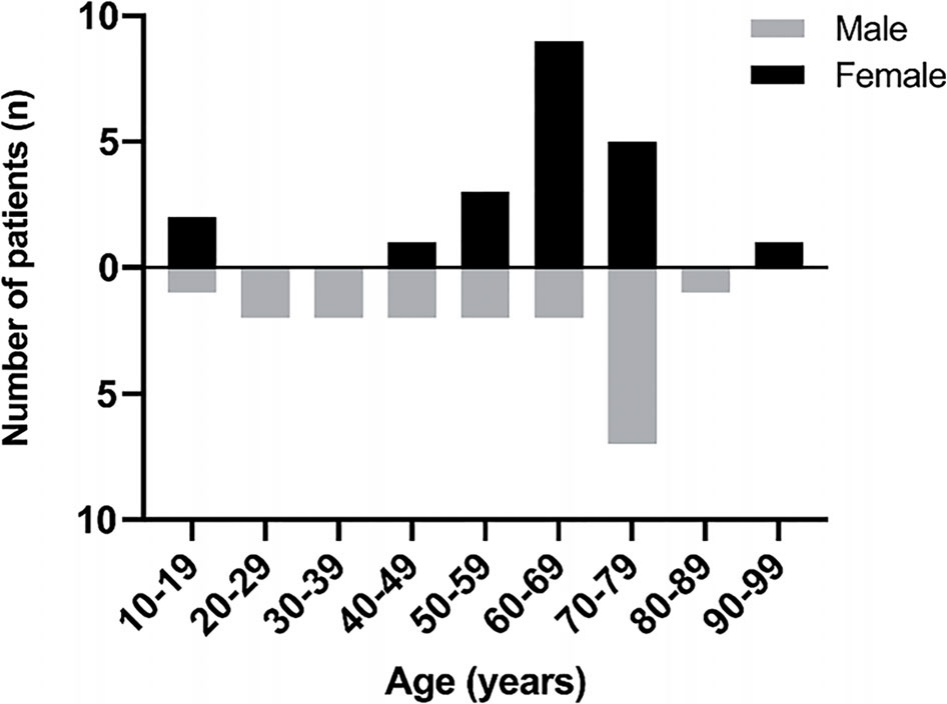
The age and sex of PPL patients.

Out of the 36 adult cases reviewed here, 19 patients (52.8%) were female and 17 patients (47.2%) were male (F: M was 1.12:1) and the mean age was found to be 58 years (mean age for males was 56 years and 61 years for females). Out of the four pediatric patients, two were of each sex (**Figure 4**).

### Tumor Location and Pathology Type

**Figure 5:**
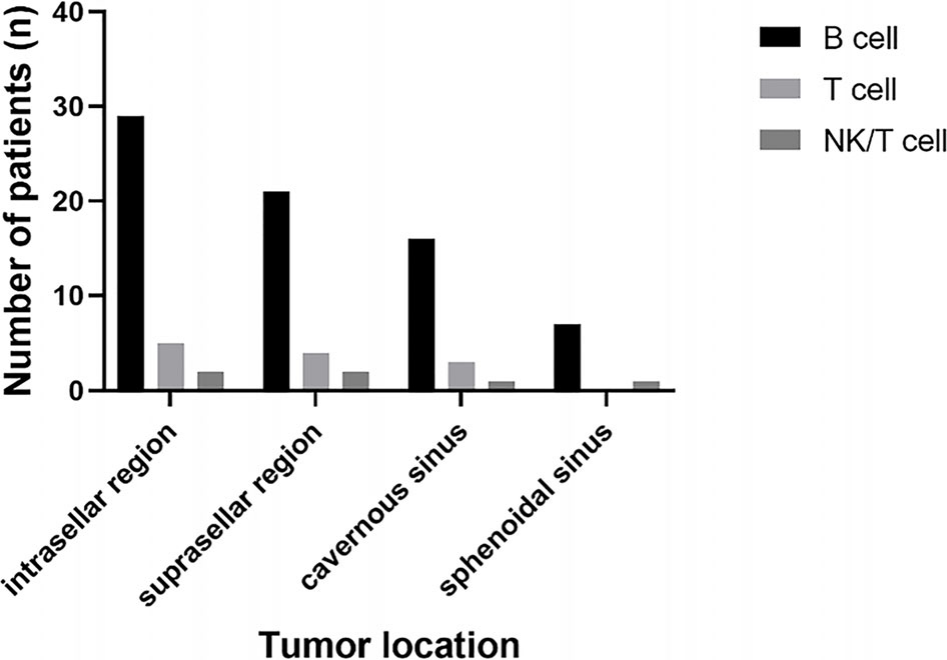
The summary of the tumor location and pathology type in PPL patients.

B cell lymphoma was the most common pathology type in PPL patients (in 32 patients, 80%), followed by T cell (in five patients, 12.5%) and NK/T cell (in two patients, 5%). Out of the five cases of T cell type, four had concomitant pituitary adenomas or B cell lymphoma, showing the tendency of T cell type PPL. Tumor location was identified through radiological findings of MRI or FDG-PET/CT, or both of them, in which 37 patients had a tumor in the intrasellar region (92.5%), 29 patients in the suprasellar region (72.5%), 21 patients in cavernous sinus (52.5%), and 11 patients in the sphenoidal sinus (27.5%) (**Figure 5**).

### Clinical Presentation

**Figure 6:**
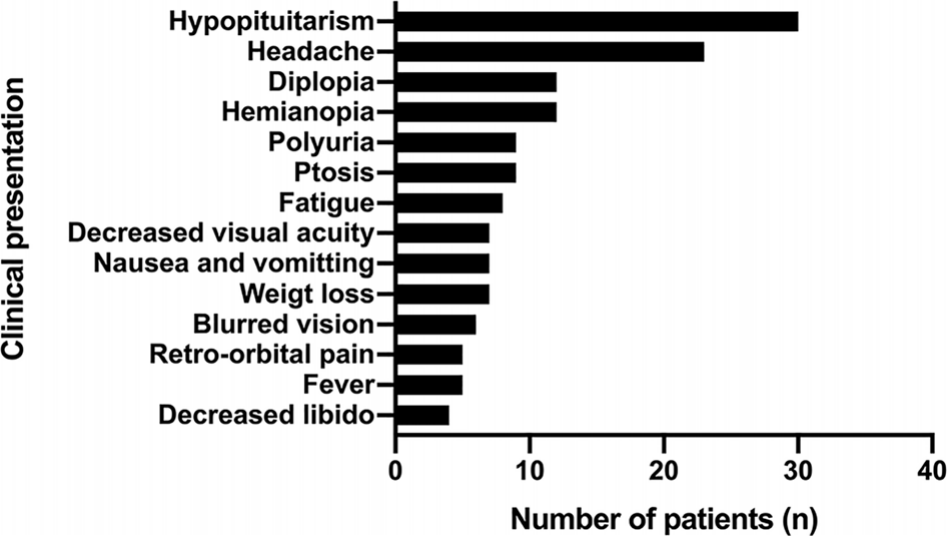
The common clinical presentations of PPL patients.

Hypopituitarism (in 30 patients, 75%) and headache (in 23 patients, 57.5%) were the most common presentations of PPL, followed by hemianopia (in 12 patients, 30%) and diplopia (in 12 patients, 30%). Other symptoms included fatigue, weight loss, fever, retro-orbital pain, nausea, and vomiting (**Figure 6**).

**Figure 7:**
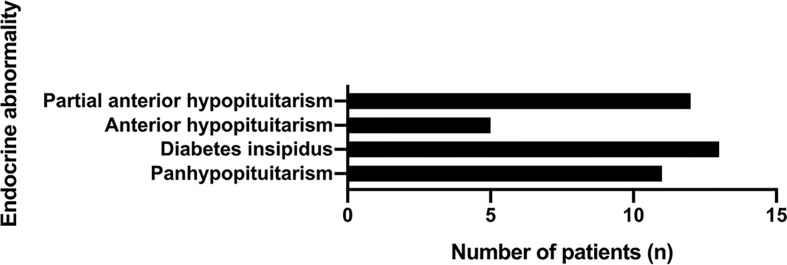
The hypopituitarism in PPL patients.

Among the patients with hypopituitarism (30 patients, 75%), panhypopituitarism was present in 11 patients (36.7%). Notably, diabetes insipidus was reported in 13 patients (43.3%). Apart from the patients concomitant with panhypopituitarism, only five patients showed complete anterior hypopituitarism (16.7%), while 12 patients showed partial anterior hypopituitarism (40%) (**Figure 7**).

### Treatment Strategies and Outcome

The therapeutic regimen for PPL is usually a combination of surgery, chemotherapy, and/or radiotherapy. Since the case reports included in our study had an unbalanced follow-up duration, it might have resulted in inevitable statistical bias and the possibility that the tumor recurrence and metastasis were neglected. Thus, we attempted to evaluate the response to treatment as “responding” or “non-responding”, where “responding” indicated that the patient had achieved remission or had stable remnant tumor without progression after the therapies were initiated whereas, the “non-responding” implied that the patient had tumor progression, death, metastasis, or relapses after the treatment was adopted.

In this review, treatment strategies were reported in 35 patients, out of which 10 patients (28.6%) received a single treatment modality while 25 patients (71.4%) received combined ones. Only one patient underwent just a resection but died soon after the surgery. Two patients received radiotherapy, but only one of them was sensitive to it, and the other showed no response to the treatment. Notably, seven patients received chemotherapy, and six of them (85.7%) turned out to be effective. In the combined treatment group, four out of five patients (80%) responded to chemotherapy and radiotherapy, six out of nine patients (66.7%) responded to resection and chemotherapy, whereas two out of four patients (50%) responded to resection and radiotherapy while five out of seven patients (71.4%) responded to resection, chemotherapy, and radiotherapy.

### Mortality

Among 40 patients of PPL, 12 of them (30%) died, where deaths were caused by the tumor (3), myocardial infarction (1), pulmonary failure (1), pneumonia (1), infection (2), and metastasis (2) while one patient died soon after the treatment, whereas the other one died before the treatment. The median mortality time was 8.7 months (P25:2 months, P75:8 months).

### Differential Diagnosis

The diagnosis of PPL was not suspected in 27 patients, and before biopsy could confirm PPL, other diagnoses were made. Pituitary adenoma was the most common in the differential diagnosis (18 cases, 66.7%), followed by lymphocytic hypophysitis (four cases, 14.8%), Langerhans cell histiocytosis (three cases, 11.1%), metastases (three cases, 11.1%), and lymphoma (three cases, 11.1%). Meningioma and germinoma were found in two cases (7.4%), while other diagnoses included sphenoid sinusitis, granulomatous disease, schwannoma, intrasellar solitary plasmacytoma, sellar/suprasellar hemangiopericytoma, and rhabdomyosarcoma of the sphenoid sinus.

## Discussion

Primary CNS lymphoma (PCNSL) in immunocompetent patients is rare and represents about 2–4% of all intracranial neoplasms and 4–6% of all extranodal lymphomas (41–43). However, in recent years, a rising incidence has been recognized, particularly in patients older than 60 years (43). Although PPL can develop in immunosuppressed individuals, it seems to be irrelevant to the immune status of PCNSL patients. Therefore, further studies are required to investigate the underlying mechanism of this pathological damage. Previous studies tend to categorize PPL as a sub-population of PCNSL, but we recommend it to be considered as a distinct entity from PCNSL. The origins of anatomic regions involved in the PPL are embryologically different from that of the cerebral parenchyma, as these anatomic structures are outside the blood–brain barrier (2).

Surprisingly, we found that both patients had infiltrative lesions in sphenoid sinus mucosa pathologically, but the sphenoid bones that composed the sellar base were visually intact during the biopsy procedure. In this review, we identified 13 patients (32.5%) with sphenoidal involvement, where seven patients (58.3%) showed no evidence of bony erosion. Notably, two patients, including the one in our case had no radiographic evidence of sphenoidal involvement but were confirmed histologically (14). Because most cases reviewed here did not undergo pathological analysis of sphenoidal mucosa, it is highly likely that there were more patients with sphenoidal involvement of PPL. Therefore, we speculated that PPL tends to spread to the sphenoidal area through vascular and bony approaches. The vascular approach was considered because a large proportion of PPL cases demonstrated sphenoidal involvement without bony erosion. Bony erosion happens in 8% of Hodgkin’s Lymphoma (44) cases and 15% in diffuse large B-cell lymphoma, which is less common at the early stage of the lymphoma system (45) but is still a viable way by which the tumor spreads to the sphenoidal area with or without visual changes in the sphenoidal bones. To the best of our knowledge, it is the first time that the characteristic of sphenoidal involvement is concluded for PPL, which might be different from other lymphomatous pathologies. Hence, we hypothesized that the biopsy of the mucosal sphenoid sinus could be an alternative to sellar biopsy, therefore, reducing the surgical risk while conducting diagnosis.

PPL is more invasive in nature when compared to other pituitary pathologies like adenomas or lymphocytic hypophysitis. It is often aggressive and may extend to the sphenoid sinus, cavernous sinus, or the floor of the third ventricle (26). PPL is usually located in the sellar or parasellar region. But we have also found the involvement of clivus and pituitary stalk (2). The correlation between tumor location and pathology type was not identified, which means that a radiological finding cannot indicate the specific pathology of lymphoma (**Figure 5**). We noticed that four patients developed metastasis after the diagnosis of PPL. The pathology of these patients did not follow the prevalence of B cell lymphoma, where three of them showed symptoms of fever. Reports of leptomeningeal dissemination, metastasis lesions in the bone marrow, liver, lung, adrenal gland, retroperitoneal lymph nodes raised the possibility that PPL might be the initial lesion location for the systemic disease (3, 18, 20, 31). Further studies are required to test and confirm this hypothesis.

PPL usually has atypical characteristics that make it difficult to distinguish from other pituitary tumors. Endocrine abnormalities and neurological deficits are found to assist tumor localization and explain some specific clinical presentations. Hypopituitarism (30 patients, 75%) and headache (23 patients, 57.5%) were the most common presentations of PPL, followed by hemianopia in 12 patients (30%) and diplopia in 12 patients (30%). Other symptoms included fatigue, weight loss, fever, retro-orbital pain, nausea, and vomiting. About 80% of the patients represented a cranial nerve deficit (II and III CN were most frequently involved), which means that it extended to the cavernous sinus (2). Hypopituitarism in PPL usually presents with various symptoms, including fatigue, muscle weakness, loss of libido, amenorrhea, thirst, and polyuria (3). Anterior hypopituitarism was present in about 70% of the cases, and more than one-third had diabetes insipidus associated with poor prognosis, which was also seen in our review (1, 2). Moreover, the brain tissues taken at the post-mortem of the PCNSL patients identified pituitary gland involvement in about 25% of cases, particularly in the posterior lobe and not the anterior one (46). Such a frequent involvement of the posterior pituitary should be paid more attention. Distinct radiological features were not identified in the sellar and suprasellar lymphomas (29). Therefore, the pathological analysis was required to confirm the diagnosis and adjust the treatment.

As for diagnosis, it seems that pituitary biopsy is currently a gold standard for confirmation. Medical history, physical exam and radiological criteria is highly unlikely to reach the correct diagnosis. Surprisingly, some biomarkers were developed for early detection of PCNSL. Royer-Perron et al. reviewed several biomarkers of PCNSL for diagnostic purpose, such as different microRNAs (miR-21, miR-19, miR-92a, etc.), which could be tested in CSF and vitreous fluid, and IL-10 in the CSF (47). Other biomarkers tested in CSF include Circulating U2 small nuclear RNA fragments, Tumor cell-free DNA, Neopterin, Osteopontin, sTACI, sBCMA, APRIL,BAFF, VSIG4, GPNMB4, and APOC2 (47–50). Based on the studies of the markers above, we believe the specific markers for PPL would be developed soon. However, these markers could not replace the value of biopsy so far and need to be evaluated within a larger patient cohort.

The specific pathogenesis of PPL is still controversial. PPL may either derive from neoplastic transformation of normal lymphocytes that enter the CNS due to inflammatory processes or from the transformation of normal resident lymphoid tissue in the CNS (2, 28). Also, some multipotent cells, i.e., chromophobes, marginal zone cells, follicular cells, folliculo stellate cells, and colony-forming units, might activate and proliferate to develop pituitary lymphoma (29). Risk factors, including AIDS and other immunodeficiency states, along with lymphocytic hypophysitis and pituitary adenomas increase the susceptibility to PPL (1). PPL may share similar pathogenesis with primary thyroid lymphomas, noted more commonly in patients with coexisting Hashimoto thyroiditis (HT) (26). The patient of case 1 had undergone partial thyroidectomy due to a thyroid nodule, but we could not confirm whether it was associated with HT and if it was possibly a potential pathogenic factor. EBV infection is another precipitating factor that induces oncogenic protein expression with subsequent loss of apoptosis and increased proliferation of lymphocytes, but both our patients were found to be EBER-ISH negative (51).

Pediatric patients with PPL seem to be different from their adult counterparts in terms of pathogenesis, clinical presentation, and radiological features. A data suggested that PPL in pediatric patients may be a different disease from that found in adults (40). They tend to have a smaller tumor size, mainly located in the pituitary stalk, which is easily misdiagnosed as germ cell tumors or Langerhans cell histiocytosis that occurs more often in children (39, 40). More importantly, these children were not likely to develop a symptom of headache. No distinct difference was observed in gender and age, and a good prognosis was achieved under chemotherapy. However, more studies are required for further investigation.

The management of PPL usually follows the protocols of treatment for PCNSL because of the lack of studies conducted on this extremely rare disease (2). The therapeutic regimen for PPL is usually a combination of surgery, chemotherapy, and/or radiotherapy (including gamma knife surgery and common radiotherapy) (5). The surgical treatment using the transsphenoidal approach may be a viable option considering the specific location and bulk mass of the PPL lesion. However, the surgical intervention suggests no obvious benefits to the outcome of PCNSL, and the main purpose of it remains to establish a diagnosis *via* biopsy (42, 52).

Chemotherapy was proven effective in the treatment of PCNSL. The therapeutic regimen consisted of high-dose chemotherapy based on methotrexate (HD-MTX) combined with rituximab and other cytostatic drugs that can penetrate the blood–brain barrier and are highly recommended if the patient’s general condition permits (53). Both our patients received chemotherapy as the first-line of treatment but with varied clinical outcomes. Case 1 patient was sensitive to chemotherapy, which was confirmed by MRI that revealed a significant reduction in the size of the sellar mass, while the Case 2 patient showed a larger sellar mass compared to the previous radiological findings. Pathologically, both our patients showed a Bcl-2, Bcl-6, and C-MYC positive pattern indicating a poor prognosis. The index of p53 was positive in both patients and was more strongly expressed in the Case 2 patient, which might have contributed to the distinction of response against chemotherapy. Notably, the Case 2 patient exhibited aberrantly high positivity of Bcl-6 (95%+) and C-MYC (80%+), also the Ki-67 index had increased up to 90%, which indicated a much poorer prognosis. Given the dominant use of methotrexate in the treatment of PPL, we speculate that the insensitivity of chemotherapy in the Case 2 patient might have resulted from drug resistance or ineffectiveness. Several alternative drugs such as pemetrexed and ibrutinib and salvage chemoimmunotherapy with rituximab, ifosfamide, and etoposide (R-IE regimen) were found to be clinically effective to some degree in relapsed or refractory PCNSL (54–56). Proper antitumor agents based on mRNA expression of drug-resistance genes in tumor tissues, including MDR-1, MRP-1, MRP-2, MXR-1, MGMT, GST-pei, and topoisomerase II alpha can also be selected accordingly (57).

Moreover, high-dose chemotherapy with autologous stem cell transplantation (HD-AST), which was first shown as an efficient therapeutic approach to recurrence, was proposed as a valuable alternative to the whole brain radiotherapy (WBRT) to consolidate the first-line of treatment (58). Two of the PPL patients reviewed in our study had received HD-AST after the occurrence of multiple relapses, but only one patient showed a good evolution (23, 26). Molecular studies are targeting PCNSL, which may be a novel therapeutic option in the future. Immune-mediated therapies and targeted therapies are under investigation and will achieve a more precise treatment with less damaging clinical outcomes (59).

PPL has a much poorer prognosis when compared to the pituitary involvement of systemic lymphoma (6). The overall mean survival rate of immunocompetent PPL patients was 14.4 months. There was no significant difference in terms of survival rates according to the adjuvant treatment strategies, although a combination of radiotherapy and chemotherapy provided a little longer mean survival rate than radiotherapy or chemotherapy alone (2). A newly developed prognostic score for PCNSL (Taipei Score) may offer disease risk stratification and facilitate clinical decision-making. Here, an age ≥80, deep brain lesions, and ECOG ≥2 were considered independent risk factors for PFS where each factor was assigned one point and there were four distinct risk groups (0–3 points) (60). Currently, the prediction of prognosis and treatment modalities of PPL follow the protocols of PCNSL due to its extreme rarity and also the scarcity of adequate research materials. Although PPL might have a different nature from PCNSL, we should also notice the similarity between these two pathologies. We believe that an exclusive prognostic score for PPL will be developed one day with more studies and cases being reported. Fortunately, with the advancing therapeutic strategies nowadays, the treatment outcomes are improving, and also PCNSL is now considered curable in some cases (59).

## Conclusion

PPL is an emerging rare clinical entity with a poor prognosis, where advanced therapeutic strategies with a more precise and less damaging effect are under investigation. We are less likely to reach a confirmative diagnosis without the pathological analysis since distinguishing PPL from other sellar tumors is difficult just through the clinical presentations or radiographic findings. Molecular studies are required in the future to investigate the pathological mechanism and establish the biomarkers for early detection and therapeutic orientation. The prognostic score for PCNSL might be a useful tool to predict the clinical outcome of PPL. Since only 40 cases are reported to date, further investigations about PPL are expected in the future, and more details about the distinction between PPL and PCNSL are yet to be studied.

## Data Availability Statement

The original contributions presented in the study are included in the article/supplementary materials. Further inquiries can be directed to the corresponding authors.

## Author Contributions

LD and JL drafted the manuscript and conducted the literature review. LC and XZ performed clinical management for patients. YZ guided the hematological treatment. BP conducted the pathological analysis. LL and HP edited the manuscript and verified the literature review. YY and HZ conceived the research idea and edited the manuscript. All authors contributed to the article and approved the submitted version.

## Funding

This work was supported by a grant from the National Key Research and Development Program of China (no. 2016YFC0901501) and CAMS Innovation Fund for Medical Science (CAMS-2016-I2M-1-002)

## Conflict of Interest

The authors declare that the research was conducted in the absence of any commercial or financial relationships that could be construed as a potential conflict of interest.
